# Enzymatic Assembly of Chitosan-Based Network Polysaccharides and Their Encapsulation and Release of Fluorescent Dye

**DOI:** 10.3390/molecules29081804

**Published:** 2024-04-16

**Authors:** Masayasu Totani, Aina Nakamichi, Jun-ichi Kadokawa

**Affiliations:** Graduate School of Science and Engineering, Kagoshima University, 1-21-40 Korimoto, Kagoshima 890-0065, Japan; m-totani@cb.kagoshima-u.ac.jp (M.T.); k6265936@kadai.jp (A.N.)

**Keywords:** α-amylase, encapsulation, enzymatic assembly, fluorescent dye, glucan phosphorylase, network polysaccharide, release, water-soluble chitosan

## Abstract

We prepared network polysaccharide nanoscopic hydrogels by crosslinking water-soluble chitosan (WSCS) with a carboxylate-terminated maltooligosaccharide crosslinker via condensation. In this study, the enzymatic elongation of amylose chains on chitosan-based network polysaccharides by glucan phosphorylase (GP) catalysis was performed to obtain assembly materials. Maltoheptaose (Glc_7_) primers for GP-catalyzed enzymatic polymerization were first introduced into WSCS by reductive amination. Crosslinking of the product with the above-mentioned crosslinker by condensation was then performed to produce Glc_7_-modified network polysaccharides. The GP-catalyzed enzymatic polymerization of the α-d-glucose 1-phosphate monomer from the Glc_7_ primers on the network polysaccharides was conducted, where the elongated amylose chains formed double helices. Enzymatic disintegration of the resulting network polysaccharide assembly successfully occurred by α-amylase-catalyzed hydrolysis of the double helical amyloses. The encapsulation and release of a fluorescent dye, Rhodamine B, using the CS-based network polysaccharides were also achieved by means of the above two enzymatic approaches.

## 1. Introduction

**Figure 1:**
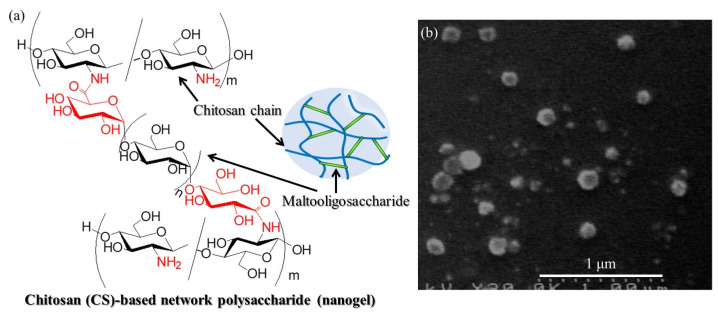
(**a**) Chemical structure and (**b**) SEM image of chitosan (CS)-based network polysaccharide as nanogel form.

Polysaccharides, one of the major classes of natural polymers [1,2], have been used for the fabrication of bio-based nanomaterials such as nanoparticles, nanofibers, and nanoscopic hydrogels (nanogels) via the regular assembly of polymeric chains because of their controlled and specific structures [3,4,5,6]. Polysaccharides are polymers comprising monosaccharide residues linked through glycosidic bonds with regular regio- and stereo-arrangements. Water-soluble polymers such as some polysaccharides and other crosslinkable macromolecules are the most common systems to form hydrogels through the chemical or physical crosslinking of polymeric chains [7]. Polymeric hydrogels exhibit no solubility in aqueous media owing to their network structures and have high water-holding capacity for developing medical applications [8,9]. Particularly, nanogels, which are hydrogels composed of submicron-sized three-dimensional polymeric networks, have increasingly attracted much attention as functional nanomaterials [10]. Polysaccharide-based nanogels have previously been fabricated via nanoscale network construction from polysaccharide chains [11]. For example, chitosan (CS)-based network polysaccharide nanogels have been prepared, where the chemical structure and nanoscale morphology in the SEM image are shown in Figure 1a,b, respectively [12]. CS is a β(1→4)-linked d-glucosamine (GlcN) polymer and has thus a number of amino groups generated by the deacetylation of *N*-acetyl-d-glucosamine units in an abundant natural polysaccharide, chitin [13]. Therefore, condensation of water-soluble chitosan (WSCS) with maltooligosaccharides with carboxylate groups at both ends (carboxylate-terminated maltooligosaccharides, GlcA-Glc*_n_*-COONa, Figure S1) as crosslinkers was conducted in the presence of a condensing agent for chemical crosslinking to construct three-dimensional polymeric networks on the nanoscale. WSCS is a relatively low-molecular-weight CS (approximately 1.0–5.0 × 10^4^), which can be prepared by the complete deacetylation of chitin under alkaline conditions and subsequent oxidative depolymerization by treatment with hydrogen peroxide (Figure S2) [14,15]. 

The preparation procedure for GlcA-Glc*_n_*-COONa has been established in previous studies by means of thermostable glucan phosphorylase (GP, isolated from thermophilic bacteria *Aquifex aeolicus* VF5)-catalyzed enzymatic α-glucuronylation using α-d-glucuronic acid 1-phosphate (GlcA-1-P) and a maltooligosaccharide with a carboxylate group (Glc*_6_*-GlcCOONa) [16] as glycosyl donor and acceptor, respectively (Figure S1); the acceptor was obtained by oxidation at the reducing end of maltoheptaose (Glc_7_) in the presence of I_2_/NaOH [17]. GP is an enzyme that catalyzes consecutive glycosylations using α-d-glucose 1-phosphate (Glc-1-P) and maltooligosaccharides as glycosyl donors and acceptors, respectively, according to enzymatic polymerization manner, to produce α(1→4)-glucan, amylose [18]; the former and latter substrates act as monomers and primers, respectively, in the polymerization. GP shows weak specificity for glycosyl donors, in which non-native sugar 1-phosphate glycosyl donors are recognized as analog substrates of native Glc-1-P, such as GlcA-1-P, in glycosylations with the maltooligosaccharide acceptor to introduce the corresponding sugar residues at the non-reducing end of the acceptor [19,20,21,22]. Subsequently, GlcA-Glc*_n_*-COONa, prepared by thermostable GP-catalyzed enzymatic α-glucuronylation using GlcA-1-P and Glc*_6_*-GlcCOONa, was used as the crosslinker for the condensation of amino groups in WSCS in the presence of a condensing agent in water to fabricate CS-based network polysaccharides.

**Figure 2:**
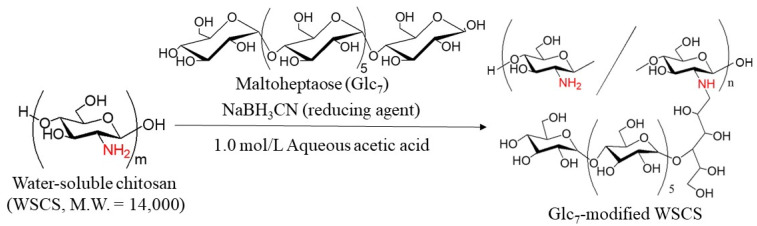
Synthesis of maltoheptaose (Glc_7_)-modified water-soluble chitosan by reductive amination.

**Figure 3:**
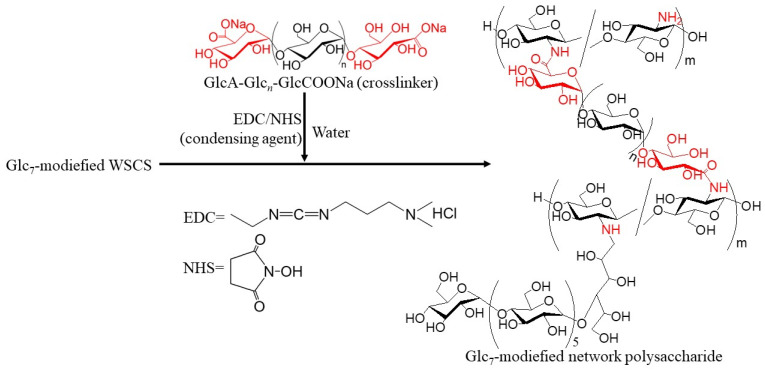
Synthesis of Glc_7_-modified network polysaccharide.

**Figure 4:**
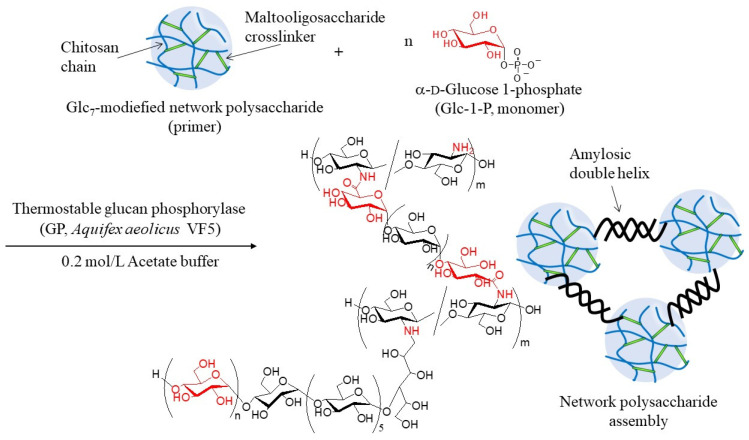
Thermostable glucan phosphorylase (GP, *Aquifex aeolicus* VF5)-catalyzed enzymatic polymerization using Glc_7_-modified network polysaccharide to obtain network polysaccharide assembly.

In this study, GP-catalyzed enzymatic assembly of CS-based network polysaccharides was attempted according to the following procedure. Glc_7_ primers for GP catalysis were introduced into WSCS by reductive amination using NaBH_3_CN as a reducing agent to produce Glc_7_-modified WSCS (Figure 2). The product was then converted into a network structure via condensation with GlcA-Glc*_n_*-COONa in the presence of a condensing agent (1-ethyl-3-(3-dimethylaminopropyl)carbodiimide hydrochloride (EDC)/*N*-hydroxysuccinimide (NHS)) (Figure 3). Thermostable GP-catalyzed enzymatic polymerization of Glc-1-P was performed using the Glc_7_ primer ends present on WSCS to elongate the amylosic chains (Figure 4). The elongated chains form well-known amylosic double helices among the network of polysaccharides to produce the assembled products. Depending on the enzymatic polymerization conditions, hydrogels and films formed from the assembled products. Disintegration of the obtained assembly was also conducted by α-amylase-catalyzed enzymatic hydrolysis of the double helical amyloses among the network polysaccharides. Accordingly, the encapsulation and release behaviors of a fluorescent dye, Rhodamine B, using CS-based network polysaccharides were investigated by GP-catalyzed assembly and α-amylase-catalyzed disintegration approaches, respectively.

## 2. Results and Discussion

**Figure 5:**
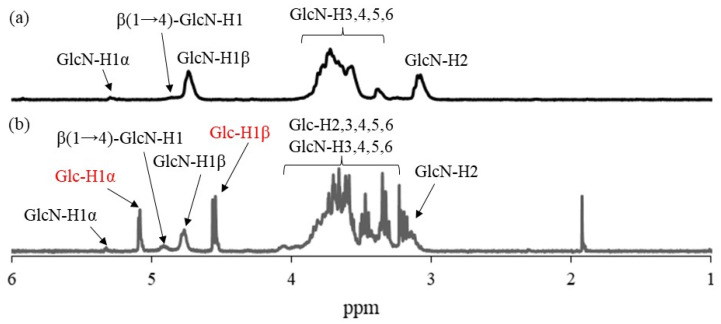
^1^H NMR spectra of (**a**) WSCS and (**b**) Glc_7_-modified WSCS (run 1) in DCl/D_2_O.

The Glc_7_ primers were first modified on WSCS, which was prepared by the complete deacetylation of chitin under alkaline conditions and subsequent oxidative depolymerization using hydrogen peroxide (Figure S2) [23] by reductive amination in the presence of NaBH_3_CN (Figure 2) [24]. The reaction was performed in two different feed ratios of amino groups in WSCS, Glc_7_, and NaBH_3_CN (1:1:5 and 1:2:5, runs 1–3, see Table 1 below) in 1.0 mol/L aqueous acetic acid for 48 h at 50 °C. Different concentrations of reaction solutions were also employed (12, 18, and 24 mmol/L GlcN units). The products were isolated as insoluble fractions in methanol. ^1^H NMR analysis was conducted in DCl/D_2_O after complete dissolution by acidic hydrolysis of glycosidic linkages in the products because the products were insoluble in common NMR solvents. Figure 5 shows the comparison of the ^1^H NMR spectra of the hydrolysate from run 1 and WSCS in DCl/D_2_O. In addition to the signals ascribed to GlcN residues detected in Figure 5a, the anomeric signals derived from Glc residues (labeled with red characters) produced by acidic hydrolysis of the Glc_7_ chains were observed at δ 4.76 and 5.08 in the ^1^H NMR spectrum of the hydrolysate (Figure 5b). This NMR result indicates the successful modification of Glc_7_ primers on WSCS by reductive amination to obtain Glc_7_-modified WSCS. The degree of substitution (DSs) of Glc_7_ with the GlcN repeating unit for the product of run 1 was calculated as 1.9% by integrating the ratio of the Glc anomeric signals to the GlcN-H2 signals. The DS values increased according to the GlcN unit concentration (2.7 and 4.2% for the products of runs 2 and 3, respectively).

**Figure 6:**
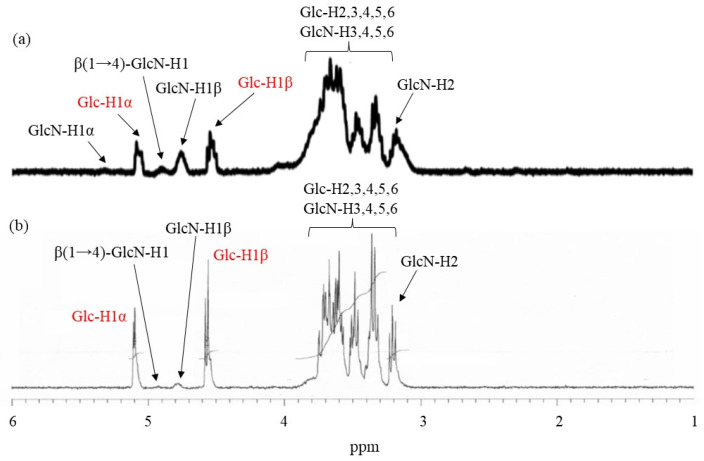
^1^H NMR spectra of (**a**) Glc_7_-modified network polysaccharide (run 1) and (**b**) network polysaccharide assembly (run 4) in DCl/D_2_O.

The residual amino groups in the obtained Glc_7_-modified WSCS were subjected to a condensation reaction with the GlcA-Glc*_n_*-COONa crosslinker in water, according to the previously reported procedure, using EDC/NHS as the condensing agent to produce Glc_7_-modified network polysaccharides (Figure 3) [12]. An aqueous solution of GlcA-Glc*_n_*-COONa, EDC, and NHS at a molar ratio of 0.5:1:1 (carboxylate/condensing agent =1:1) was added to an aqueous solution of Glc_7_-modified WSCS (runs 1–3) at a molar ratio of carboxylate to amino groups of 0.05:1, and the mixture was kept for 7 h at room temperature. The products were isolated as insoluble fractions in methanol. The ^1^H NMR spectrum of the product obtained using Glc_7_-modified WSCS from run 1, which was prepared by acid hydrolysis and dissolution in DCl/D_2_O (Figure 6a), indicated a slight increase in the integrated ratio of the Glc anomeric signals (labeled by red characters) to the GlcN anomeric signals compared with that of Glc_7_-modified WSCS (Figure 5b). These data suggest the presence of crosslinker chains composed of Glc residues via a condensation reaction with amino groups in WSCS. For example, the ratio of Glc_7_ to the GlcN repeating unit was calculated to be 1.1% using ^1^H NMR analysis.

**Figure 7:**
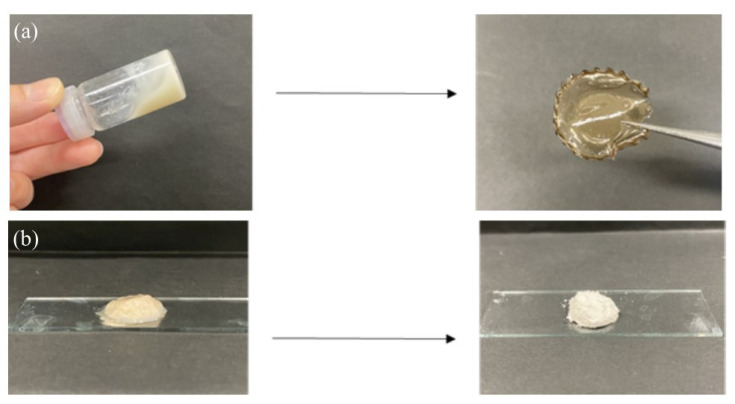
Formation of (**a**) film and (**b**) hydrogel from network polysaccharide assemblies.

The thermostable GP-catalyzed enzymatic polymerization of Glc-1-P was then performed with the Glc_7_ primers (DSs = 1.2, 2.7, and 4.2%, runs 1–3) on the obtained network polysaccharides in 0.2 mol/L acetate buffer for 48 h at 50 °C to assemble them via amylosic double helix formation (Figure 4). Varying Glc-1-P (monomer)/Glc_7_ (primer) feed ratios were employed, as listed in Table 2, because this ratio regulates the molecular weight of the enzymatically produced amylose. As the reaction mixtures at a low feed ratio (i.e., 100) maintained the solution state as polymerization progressed (runs 4, 7, and 8), the products were isolated by dialysis and lyophilization of the reaction mixtures. With an increase in the feed ratio (runs 5, 6, and 9), the reaction mixtures became viscous. Consequently, the reaction mixture turned into a gel at a feed ratio of 500, with a larger DS value of Glc_7_ (4.2%, run 10). These viscous and gel products were washed with water and lyophilized for purification and isolation. Furthermore, films were formed by casting and drying DMSO solutions of these products, and treatment of the gelling reaction mixture of run 10 with water produced a self-standing hydrogel, as shown in Figure 7a,b, respectively. Viscous and gel mixtures were produced by GP-catalyzed enzymatic polymerization in the higher monomer (Glc-1-P)/primer (Glc_7_) feed ratios and the larger DS values of the Glc_7_ primers on WSCS; amyloses with higher molecular weights and larger assembly densities were produced via amylosic double helix formation. The ^1^H NMR spectroscopic pattern of the samples prepared by acidic hydrolysis and dissolution in DCl/D_2_O, representatively shown as the spectrum of the sample from the product of run 4 (Figure 6b), is similar to that of the Glc_7_-modified network WSCS (Figure 6a); however, the intensities of the Glc anomeric signals (labeled with red characters) to the GlcN anomeric signals in the former spectrum were much higher than those of the latter. The NMR results strongly supported the progress of GP-catalyzed enzymatic polymerization to introduce amylose chains into the network of polysaccharides.

**Figure 8:**
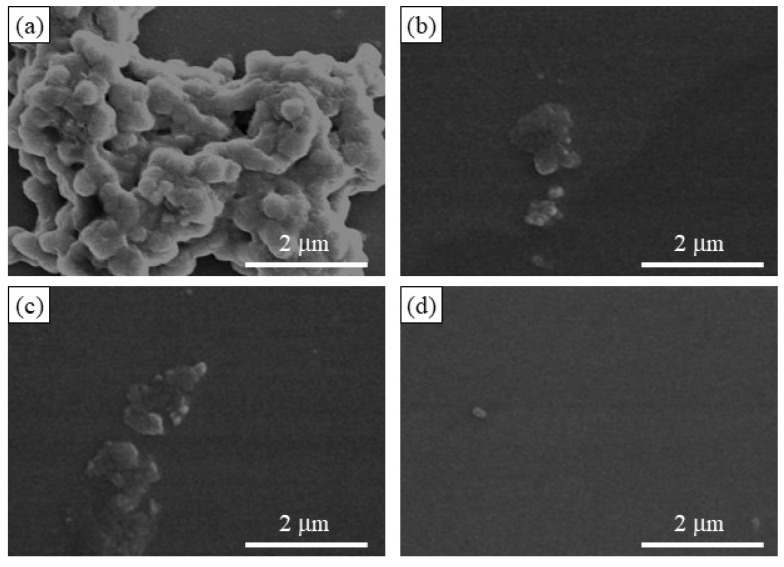
SEM images of (**a**) network polysaccharide assembly (run 4) and (**b**–**d**) reaction mixtures by α-amylase treatment for 2, 4, and 6 h.

**Figure 9:**
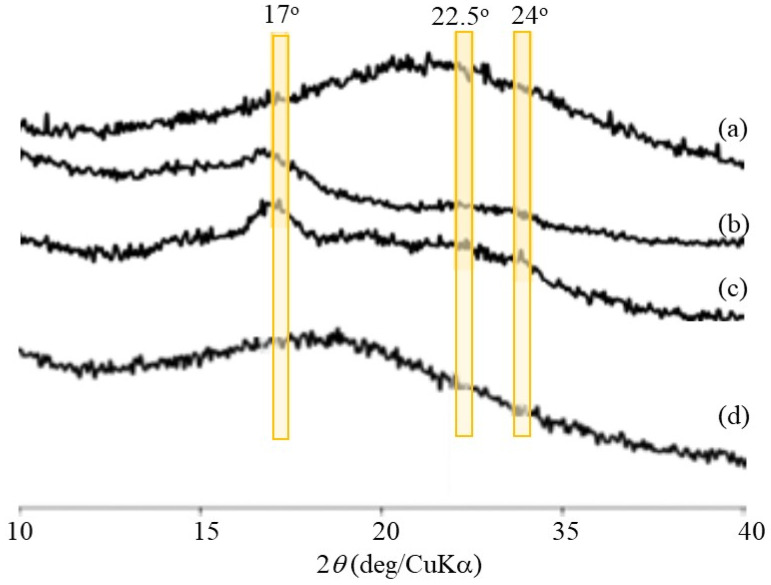
XRD profiles of (**a**) Glc_7_-modified WSCS, (**b**) amylose, (**c**) network polysaccharide assembly (run 4), and (**d**) product by α-amylase treatment for 48 h.

The SEM image of the enzymatic polymerization product (run 4, Figure 8a) demonstrates the assembling morphology of nanoparticles, suggesting that the elongated amylose chains form double helices among the network polysaccharides; the SEM image of the network polysaccharide nanoparticles is shown in Figure 1b. Powder X-ray diffraction (XRD) measurements were conducted to confirm the presence of a double helix structure in the polysaccharide network assembly. The XRD profile of the Glc_7_-modified WSCS did not exhibit any diffraction pattern owing to its amorphous nature (Figure 9a). In contrast, the XRD profile of the enzymatic polymerization product (run 4) in Figure 9c clearly shows a typical diffraction pattern (17°, 22.5°, and 24°) assignable to the amylosic double helical crystalline structure, similar to that of a pure amylose sample (Figure 9b). These XRD results indicate that the CS-based network polysaccharides were efficiently assembled via double helix formation by the amylose chains during GP-catalyzed enzymatic polymerization.

Accordingly, disintegration of the network polysaccharide assembly (run 7) was attempted by α-amylase-catalyzed enzymatic hydrolysis of the double helical amyloses; this is an exo-enzyme that catalyzes random hydrolysis of α-glucan chains, i.e., amylose to produce disaccharides and maltoses. The network polysaccharide assembly (run 7) was treated with α-amylase (from *Bacillus amyloliquefaciens*) in 0.2 mol/L acetate buffer at 40 °C, and the reaction mixtures were taken every 2 h. The SEM images of the spin-coated samples clearly show the gradual disintegration of the assembly morphology with prolonged reaction times (Figure 8b,c). After 6 h of enzymatic treatment, the morphology of the individual nanoparticles was observed using SEM (Figure 8d). The XRD profile of the sample obtained by dialyzing the reaction mixture after 48 h did not exhibit any amylosic double helical diffraction pattern (Figure 9d), which was different from that before enzymatic treatment (Figure 9c). The above results suggest that α-amylase-catalyzed enzymatic hydrolysis of the double helical amyloses efficiently occurred to disintegrate the network polysaccharide assembly.

**Figure 10:**
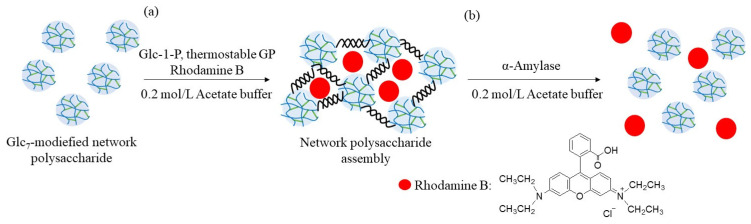
(**a**) Encapsulation and (**b**) release experiments of Rhodamine B using network polysaccharide assembly.

The encapsulation and release behaviors of the fluorescent dye were investigated using the above enzymatic assembly and disintegration processes (Figure 10). Rhodamine B was selected as the fluorescent dye because it has the ability to form electrostatic interactions with polysaccharides with amino groups (such as WSCS), owing to the presence of an acidic carboxylic acid group, as shown in Figure 10. Therefore, the abovementioned enzymatic assembly experiment using the Glc_7_-modified network polysaccharide of run 7 by thermostable GP-catalyzed enzymatic polymerization was performed in the presence of Rhodamine B (0.2 equiv. with residual amino groups in WSCS) in 0.2 mol/L acetate buffer for 48 h at 50 °C (Figure 10a). The reaction mixture was dialyzed, and the concentrated dialysate was lyophilized to give the assembling product encapsulating Rhodamine B. The amount of dye present in the outer filtrate was calculated by its fluorescence spectrum when excited at 555 nm. Therefore, the amount of the encapsulated dye in the assembling product was 38% (2.2 mg/6.0 mg), owing to electrostatic interactions between the amino and carboxylic acid groups.

**Figure 11:**
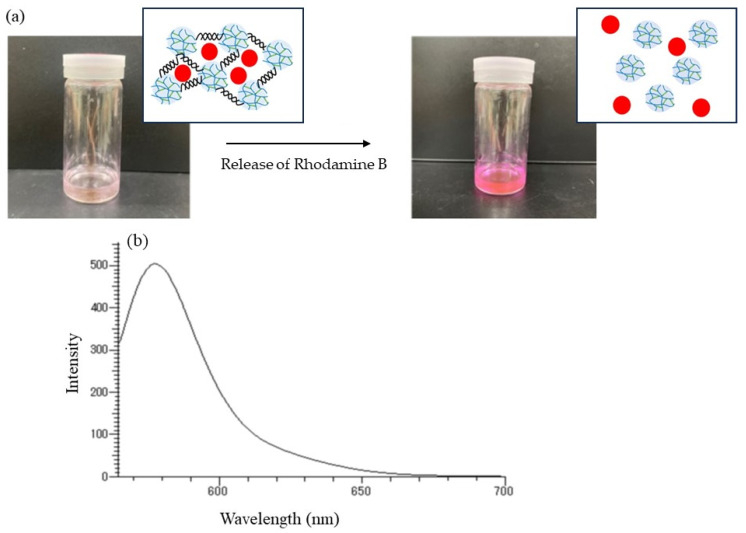
(**a**) Color change in α-amylase treatment mixture using encapsulated material in 0.2 mol/L acetate buffer for 4 h and (**b**) its fluorescence spectrum excited at 550 nm.

The release behavior of the encapsulated Rhodamine B was then explored by α-amylase-catalyzed enzymatic disintegration of the network polysaccharide assembly. When the mixture of the assembly with 0.2 mol/L acetate buffer was stirred in the presence of α-amylase (from *Bacillus amyloliquefaciens*) for 4 h at 40 °C (Figure 10b); optimal temperatures for hydrolysis of soluble starch by α-amylase from this source were reported to be 40–60 °C [25], the color gradually changed to red, as shown in Figure 11a, indicating that Rhodamine B was released into the acetate buffer by enzymatic disintegration. A fluorescence peak at 575 nm, assigned to Rhodamine B, was then detected in the fluorescence spectrum of the reaction mixture excited at 550 nm (Figure 11b).

## 3. Materials and Methods

### 3.1. Materials

Thermostable GP (isolated from *Aquifex aeolicus* VF5) was provided by Dr. Takeshi Takaha (Sanwa Starch Co., Ltd., Nara, Japan [26]). Chitin powder from crab shells was purchased from FUJIFILM Wako Pure Chemical Corporation (Osaka, Japan). GlcA-1-P was prepared by TEMPO-mediated oxidation of Glc-1-P according to a previously reported procedure [27]. Glc_7_ was synthesized by selective hydrolysis of the glycosidic bond of β-cyclodextrin under acidic conditions [28]. All other reagents and solvents were used as received from commercial sources.

### 3.2. Synthesis of GlcA-Glc_n_-GlcCOONa (Figure S1) [16]

A solution of I_2_ in methanol (3.62 g/24.4 mL) was mixed with an aqueous solution of Glc_7_ (5.70 g, 4.90 mmol/10 mL) and stirred at 40 °C for 2 h. After the dropwise addition of a solution of NaOH in methanol (2.66 g/75.5 mL), the obtained mixture was stirred for 1 h at 40 °C. The precipitate was isolated via decantation and dried under reduced pressure for 10 h at room temperature. A solution of the product in water (40 mL) was treated with activated carbon for 1 h at room temperature while stirring. After the activated carbon was filtered, the filtrate was concentrated and lyophilized to yield crude Glc*_6_*-GlcCOONa (2.38 g).

A mixture of Glc*_6_*-GlcCOONa (0.100 g, 0.084 mmol) and GlcA-1-P (0.428 g, 1.26 mmol) in 0.2 mol/L sodium acetate buffer (6.0 mL, pH 6.2) was incubated with thermostable GP (30 U) for 48 h at 50 °C. The reaction solution was concentrated and subjected to an anion exchange column (Amberlite IRA 400 J Cl, Cl^−^ form, eluent: 1.0 mol/L aqueous acetic acid), and the fractions containing the products were collected and neutralized with 2.0 mol/L aqueous Na_2_CO_3_. The obtained solution was dialyzed against water (molecular cut off: 1000) and concentrated at approximately 70 °C. After the insoluble deactivated enzyme was removed using cotton plug filtration, the filtrate was lyophilized to give GlcA-Glc*_n_*-GlcCOONa (0.0221 g, 0.016 mmol) with a yield of 19.0%. ^1^H NMR (D_2_O) δ 3.49 (t, *J* = 9.6 Hz, GlcA-H4‴), 3.57–4.03 (m, GlcA-H2,3,5, Glc-H2,3,4,5,6, GlcCOONa-H4,5,6), 4.11–4.13 (m, GlcCOONa-H2,3), and 5.17 (m, Glc-H1′), 5.41 (m, Glc-H1″, GlcA-H1‴).

### 3.3. Preparation of WSCS (Figure S2) [23,29]

A mixture of chitin (10.0 g, 0.049 mol) and 40 wt% aqueous NaOH (500 mL) was stirred for 7 h at 160 °C. The residue was isolated via filtration and washed with water until the filtrate became neutral. After the product was mixed with 10 wt% aqueous acetic acid (500 mL) and left to stand for 12 h at room temperature, the residue was filtered. The filtrate was mixed with a NaOH pellet (85.0 g), and the resulting swollen precipitate was isolated by filtration. A mixture of the product and 40 wt% aqueous NaOH (500 mL) was stirred for 12 h at 160 °C. The residue was isolated via filtration and washed with water until the filtrate became neutral. The product was dried under reduced pressure for 8 h at 70 °C to give fully deacetylated chitosan (5.95 g, 0.0370 mol).

The obtained chitosan (1.0 g, 6.2 mmol) was solubilized in 2.0 wt% aqueous acetic acid (20 mL) by stirring for 6 h at room temperature. After 30 wt% aqueous H_2_O_2_ (5.0 mL) was added to the solution, the mixture was stirred for 4 h at 50 °C. The resulting mixture was then neutralized with 10 wt% aqueous NaOH. The resulting residue was removed by centrifugation, and the supernatant was dialyzed against water (molecular cut-off: 1000). The precipitate obtained during dialysis was removed by centrifugation, and the supernatant was lyophilized to obtain WSCS (0.0373 g, 2.3 mmol) with a yield of 37.3%. ^1^H NMR (D_2_O) δ 2.89 (br s, H2), 3.63–3.96 (m, H3,4,5,6), and 4.87 (br s, H1). The viscosity average molecular weight (*M*_v_) of the obtained water-soluble chitosan was estimated to be 14,000 using a previous method.

### 3.4. Synthesis of Glc_7_-Modified WSCS 

The typical experimental procedure was as follows (run 1). After ultrasonication of a mixture of WSCS (40.0 mg, 0.24 mmol for amino groups), Glc_7_ (553 mg, 0.48 mmol), and NaBH_3_CN (75.4 mg, 1.2 mmol) with 1.0 mol/L aqueous acetic acid (20 mL), the resulting solution was stirred for 72 h at 50 °C. The reaction mixture was then poured into methanol (300 mL) to precipitate the product. The precipitate was isolated by filtration, washed with methanol, and dried under reduced pressure for 10 h at room temperature to produce Glc_7_-modified WSCS (140 mg). ^1^H NMR (DCl/D_2_O) δ 3.12 (m, GlcN-H2), 3.84–3.23 (m, Glc-H2,3,4,5,6, GlcN-H3,4,5,6), 4.54 (m, GlcH-1β), 4.76 (m, GlcN-H1β), 4.92 (m, β(1→4)-GlcN-H1), 5.08 (m, Glc-H1α), and 5.32 (m, 0.11H, GlcN-H1α).

### 3.5. Synthesis of Glc_7_-Modified Network Polysaccharide

A solution of GlcA-Glc*_n_*-GlcCOONa (5.9 mg, 4.02 μmol, COONa group; 8.05 μmol), EDC (1.54 mg, 8.00 μmol), and NHS (0.93 mg, 8.00 μmol) in water (0.20 mL), which was prepared by standing the mixture for 1 h at room temperature, was mixed with an aqueous solution of Glc_7_-modified WSCS (run 1, 30 mg, 0.161 mmol/0.70 mL). The obtained mixture was left to stand for 7 h at room temperature to allow condensation to progress. The reaction mixture was then poured into methanol (30 mL) to produce the precipitate. The precipitated product was isolated by filtration and lyophilized to obtain a network polysaccharide (20.2 mg). ^1^H NMR (DCl/D_2_O) δ 3.09 (br s, GlcN-H2), 3.27–3.82 (m, Glc-H2,3,4,5,6, GlcN-H3,4,5,6), 4.54 (m, Glc-H1β), 4.75, (m, GlcN-H1β), 4.87 (m, β(1→4)-GlcN-H1), 5.07 (m, Glc-H1α), and 5.32 (m, GlcN-H1α).

### 3.6. Thermostable GP-Catalyzed Enzymatic Polymerization Using the Glc_7_-Modified Network Polysaccharide

The typical experimental procedure was as follows (run 4). A mixture of the Glc_7_-modified network polysaccharide obtained by the procedure described in Section 3.5 (20 mg, 1.9 μm for Glc_7_ primer), Glc-1-P (57.8 mg, 0.19 mmol), and thermostable GP (*Aquifex aeolicus* VF5, 12 U) in 0.2 mol/L acetate buffer (2.0 mL) was stirred for 48 h at 50 °C. After the reaction mixture was dialyzed (molecular cut-off: 1000) for 12 h, the obtained insoluble material was removed by centrifugation, and the dialysate was lyophilized to give the assembled product (25.9 mg). ^1^H NMR (DCl/D_2_O) δ 3.18 (m, GlcN-H2), 3.75–3.23 (m, Glc-H2,3,4,5,6, GlcN-H3,4,5,6), 4.55 (m, Glc-H1β), 4.78, (m, GlcN-H1β), and 5.10 (m, GlcN-H1α).

When the reaction mixtures became viscous or gelled, fractions insoluble in water, which were obtained by washing them with water, were lyophilized to give the assembled products.

### 3.7. Amylase-Catalyzed Enzymatic Disintegration of the Assembled Network Polysaccharide

A mixture of the network polysaccharide assembly (run 7, 15 mg) and α-amylase (from *Bacillus amyloliquefaciens*, 15 U) in 0.2 mol/L acetate buffer (5.0 mL) was stirred at 40 °C. The reaction mixture was collected every 2 h and analyzed using SEM. After 48 h, the reaction mixture was heated for 5 h at 100 °C to deactivate the enzyme. The deactivated enzyme was filtered, and the filtrate was lyophilized. The residue was dialyzed (molecular cut-off: 1000) and the dialysate was lyophilized to obtain the sample for XRD.

### 3.8. Encapsulation of Rhocamine B 

A mixture of Glc_7_-modified network polysaccharides prepared using Glc_7_-modified WSCS from run 2 (15 mg), Glc-1-P (544 mg, 0.179 mmol), Rhodamine B (6.0 mg, 0.013 mmol, 0.2 equiv. with residual amino groups in WSCS), and thermostable GP (12 U) was stirred for 48 h at 40 °C. After the reaction mixture was dialyzed (molecular cut-off: 1000) for 5 d, the dialysate was lyophilized to yield the Rhodamine B-encapsulated product (22.2 mg).

### 3.9. Release of Rhodamine B by α-Amylase Treatment

A mixture of the Rhodamine B-encapsulated network polysaccharide assembly (22.2 mg) and α-amylase (*Bacillus amyloliquefaciens*, 8.4 U) in 0.2 mol/L acetate buffer (5 mL) was stirred for 4 h at 40 °C. The reaction mixture was then subjected to fluorescence measurements.

### 3.10. Measurements

^1^H NMR spectra were recorded using a JEOL ECX400 spectrometer (JEOL, Akishima, Tokyo, Japan). SEM images were obtained using a Hitachi S-4100H electron microscope (Hitachi High-Technologies Corporation, Tokyo, Japan) at an accelerating voltage of 5 kV. Powder XRD measurements were performed using a PANalytical X’Pert Pro MPD instrument (PANalytical B.V., Almelo, The Netherlands) with Ni-filtered Cu-Kα radiation (λ = 0.15418 nm). Fluorescence spectra were recorded on an FP-6300Q3 spectrometer (JASCO Corporation, Hachioji, Tokyo, Japan).

## 4. Conclusions

In this study, the assembly of CS-based network polysaccharides was attempted via the GP-catalyzed enzymatic polymerization of Glc-1-P. The Glc_7_ primers were first introduced into WSCS by reductive amination, followed by crosslinking with GlcA-Glc*_n_*-GlcCOONa via a condensation reaction in the presence of a condensing agent to obtain Glc_7_ primer-modified network polysaccharides. The amylose chains elongated from the primers on the products via GP-catalyzed enzymatic polymerization formed double helices among the network polysaccharides to produce the assembling materials. The appearance of the reaction mixtures changed from a solution to viscous liquid and gelled states, which were likely affected by the molecular weights of the elongated amylose chains and the assembly density, depending on the Glc_7_ primer monomer (Glc-1-P)/ primer (Glc_7_) feed ratios and the DS values of the Glc_7_ primers on WSCS. The α-amylase-catalyzed disintegration of the resulting assembly was achieved via hydrolysis of the double helical amyloses. The enzymatic assembly and disintegration of the network polysaccharides were employed for the encapsulation and release of Rhodamine B. These processes have the potential to be applied in drug delivery systems using practical drugs in the future. In addition, the present processes for the production of the network materials entirely comprising bio-based components, such as polysaccharides, induce minimum use of organic solvents and are, therefore, considered to result in low environmental load. For such practical application in the medical field, additional investigations, such as cytotoxicity of the present materials and applicability to hydrophobic and noncharged drugs, are necessary, which will be reported in forthcoming papers.

## Figures and Tables

**Table 1:** Synthesis of Glc_7_-modified WSCS ^(a)^.

Run	Molar Ratio (NH_2_:Glc_7_:NaBH_3_CN)	Concentration of GlcN Unit in 1.0 mol/L Aqueous Acetic Acid (mol/L)	DS of Glc_7_^(b)^
1	1:2:5	12	1.9
2	1:1:5	24	2.7
3	1:2:5	18	4.2

^(a)^ Reaction was carried out in 1.0 mol/L aqueous acetic acid at 50 °C for 48 h. ^(b)^ DS = degree of substitution. Determined by ^1^H NMR spectra after acid hydrolysis of the products in DCl/D_2_O.

**Table 2:** Thermostable GP-catalyzed enzymatic polymerization using Glc_7_-modified network polysaccharide to produce network polysaccharide assembly ^(a)^.

Run	Glc_7_-Modified WCSC	Feed Ratio (Glc_7_ Primer/Glc-1-P)	Appearance
4	run 1	1:100	solution
5	run 1	1:500	viscous liquid
6	run 1	1:800	viscous liquid
7	run 2	1:100	solution
8	run 3	1:100	solution
9	run 3	1:300	viscous liquid
10	run 3	1:500	gel

^(a)^ Reaction was carried out in the presence of thermostable GP (12 U) in 0.2 mol/L aqueous acetic acid at 50 °C for 48 h.

## Data Availability

Data are contained within the article and Supplementary Materials.

## Supplementary Materials

The following supporting information can be downloaded at https://www.mdpi.com/article/10.3390/molecules29081804/s1, Figure S1: Synthesis of carboxylate-terminated maltooligosaccharides (GlcAGlc*_n_*-GlcCOONa); Figure S2: Preparation of water-soluble chitosan (WSCS).
