# Supplementary material for: Enzymatic Assembly of Chitosan-Based Network Polysaccharides and Their Encapsulation and Release of Fluorescent Dye
Source: Molecules. 2024 Apr 16;29(8):1804. doi: 10.3390/molecules29081804 (PMC11052119; doi:10.3390/molecules29081804)
Supplement: Supplementary file 1 [file molecules-29-01804-s001.zip › molecules-2969760-supplementary.pdf]

Supporting Information

Enzymatic assembly of chitosan-based network polysaccharides and their encapsulation and release of fluorescent dye

Masayasu Totani, Aina Nakamichi, and Jun-ichi Kadokawa*

Graduate School of Science and Engineering, Kagoshima University, 1-21-40 Korimoto, Kagoshima 890-0065, Japan

* Corresponding author: E-mail: kadokawa@eng.kagoshima-u.ac.jp

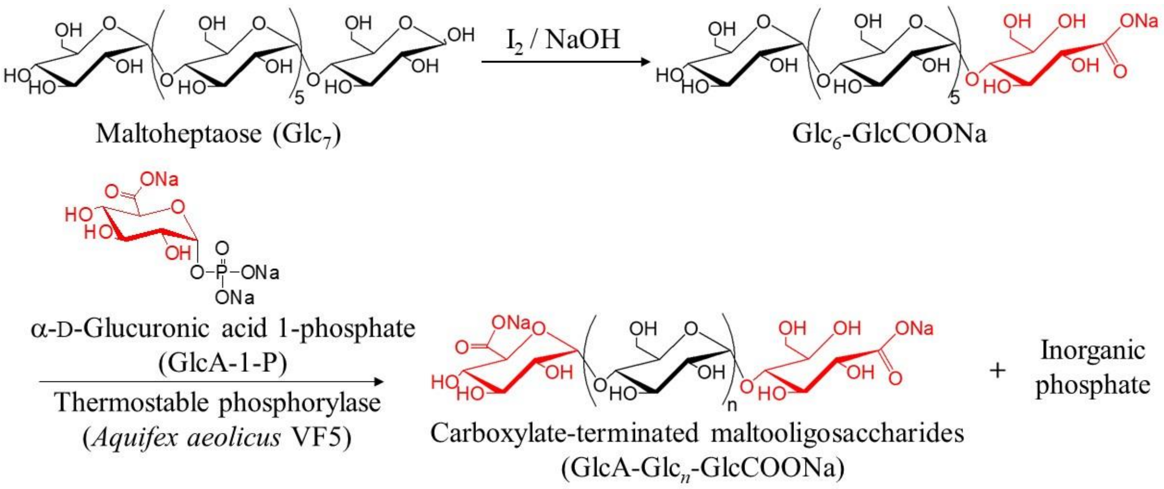

Figure S1. Synthesis of carboxylate-terminated maltooligosaccharides (GlcAGlc$_n$-GlcCOONa).

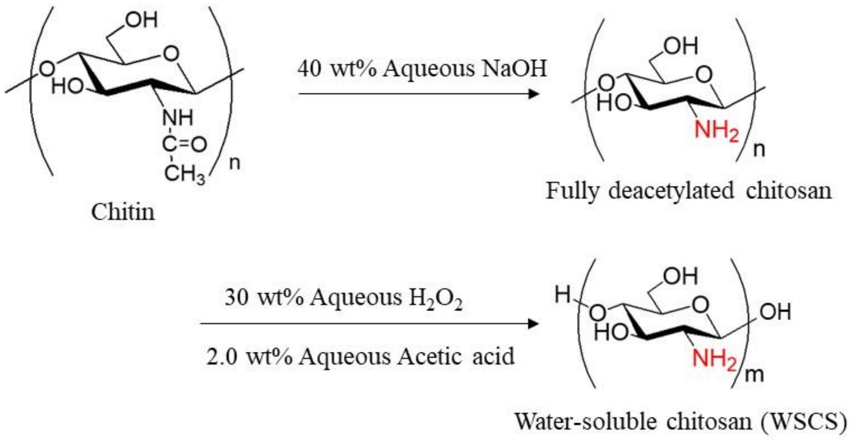

Figure S2. Preparation of water-soluble chitosan (WSCS).
